# Editorial: Insights in T Cell Biology: 2021

**DOI:** 10.3389/fimmu.2022.1039602

**Published:** 2022-09-21

**Authors:** Loretta Tuosto

**Affiliations:** Department of Biology and Biotechnology Charles Darwin, Laboratory affiliated to Istituto Pasteur Italia-Fondazione Cenci Bolognetti, Sapienza University, Rome, Italy

**Keywords:** thymus microenvironment, TCR repertoire, CD8^+^ effector T cells, lipid metabolism, heart failure

In the last decade, remarkable progresses have been made in order to elucidate the development, activation and functions of T cells in health and disease. This Research Topic (RS) contains 6 articles from editorial board members that shed light on the progress made on some crucial aspects of the T cell biology and on its future challenges.

T cell development occurs in the thymus, a primary lymphoid organ that originates from the 3^rd^ pharyngeal pouches during embryogenesis (1). The recruitment of early thymus progenitors as well as the stages of T cell maturation and selection are finely regulated by the thymus microenvironment, through the well-known interactions between developing thymocytes and thymus epithelial cells (TECs). As nicely discussed in the review by Bhalla et al., thymopoiesis is also regulated by other non-haemopoietic cells, such as neural crest cell-derived mesenchymal (Mes) cells and endothelial cells, which contribute to the formation of the thymus structure, microenvironment and vasculature. In particular, the authors discuss the role of Mes cells in the development of embryonic thymus by highlighting their ability to differentiate in pericytes, vascular smooth cells and fibroblasts as well as the role of fibroblasts in regulating TEC development (2) and central tolerance (3). In addition to their pivotal role in forming the thymus vasculature, the authors also review recent data showing the ability of some endothelial cells to differentiate in TECs and to regulate thymocyte homing as well as T cell migration and egress. As outlined by the authors, the key contribution of these cell types to thymopoiesis will be helpful for the development of personalized thymus regeneration approaches.

During thymic development and maturation, the TCR polypeptide chains undergo somatic V(D)J recombination (4), a process that theoretically yield more than 10^13^ different TCRs (TCR repertoire) (5, 6). This diversified TCR repertoire is required to ensure the recognition by T cells of the myriad of antigens from pathogenic microbes to endogenous cancers. After a nice and exhaustive description of the mechanisms and factors affecting the generation and changes of TCR diversity as well as the conventional sequencing methods used for quantitative measurement of the TCR repertoires, Katayama et al. survey the application of machine data analysis (ML) and deep learning (DL) algorithm to TCR repertoire analysis. The use of ML-based TCR repertoire analysis in COVID-19 patients and its potential applications as a diagnostic tool for several other diseases including autoimmune diseases or cancer are also discussed. In this context, by analysing and comparing the diversity and clonality of the TCRVβ repertoire in the peripheral blood of healthy donors and cancer patients, Zhuo et al. discover that the TCRβ diversity index declines in both elder individuals and cancer patients compared to younger healthy donors. Moreover, they also report that elder individuals and cancer patients have a high number of large TCR clones and lose the majority of shared common TCRVβ clones, thus strengthening the value of TCR repertoire analysis as a diagnostic tool in clinical practice.

Antigen recognition by TCR on naïve T cells leads to their activation and differentiation into memory cells, which acquire a faster and more vigorous response to pathogens. The advent of single cell resolution techniques and genome-scale epigenetic profiling revealed the heterogeneity of the memory T-cell compartment, with the self-renewing T memory stem cells (T_SCM_) that generate both long-lived T central memory cells (T_CM_), short-lived T effector memory cells (T_EM_) and T resident memory cells (T_RM_) (7). In the review by La Manna et al., the authors discuss the most recent advances in characterizing these memory subsets by focusing on CD8^+^ T cells. After an historical overview on the immunophenotype and functions of T_SCM_, T_EM_ and T_RM_ cells, the authors nicely discuss their role in infections, including SARS-CoV-2, as well as in chronic and autoimmune diseases by also highlighting the metabolic signature of each population and its impact in the maintenance of distinct CD8^+^ T-cell memory subsets.

In addition to fight pathogens and cancer cells, CD8^+^ T cells have been recently implicated in cardiac failure (8). By using single-cell RNA sequencing (scRNAseq) techniques, Komai et al. report an interesting interplay between immune cells and cardiac muscle cells in the transverse aorta constriction (TAC) murine model of heart failure. The authors show that CD8^+^ T cells are involved in the suppression of the early protective phase of hearth hypertrophy by affecting the phenotype of both resident and recruited macrophages after TAC. Of note, in CD8^+^ T cell-depleted TAC mice, the numbers of M2-like macrophages producing tissue-repairing factors increase and correlate with the acquisition by cardiomyocytes of protective metabolic and translational signatures. These novel data suggest CD8^+^ T cells as potential targets for preventing heart-destructive cardiomyocyte hypertrophy.

This first edition of the Research Topic is also enriched by an interesting bibliometric analysis of lipid metabolism in T cells from 1985 to 2022. In T cells, lipids serve as energy metabolic sources and as key signalling intermediates orchestrating the activation, differentiation and functions of distinct T cell subsets (9). By analysing 194 research articles and 96 reviews, Chen et al. show a rapid and progressive increase in researches on lipid metabolism in Th1, Th17, T regulatory cells (Treg) and CD8^+^ T cells and identified *Frontiers in Immunology* and *Nature* as the two most prolific and cited journal in the field.

## Author contributions

The author confirms being the sole contributor of this work and has approved it for publication.

## Funding

LT is funded by FISM - Fondazione Italiana Sclerosi Multipla – FISM 2020-R-Single/001 and Sapienza University of Rome “Progetto Ateneo”.

## Conflict of interest

The author declares that the research was conducted in the absence of any commercial or financial relationships that could be construed as a potential conflict of interest.

## Publisher’s note

All claims expressed in this article are solely those of the authors and do not necessarily represent those of their affiliated organizations, or those of the publisher, the editors and the reviewers. Any product that may be evaluated in this article, or claim that may be made by its manufacturer, is not guaranteed or endorsed by the publisher.
